# Geometric-phase-based shearing interferometry for broadband vortex state decoding

**DOI:** 10.1038/s41598-022-07083-w

**Published:** 2022-02-22

**Authors:** Ziyao Lyu, Changshun Wang

**Affiliations:** State Key Laboratory of Advanced Optical Communication Systems and Networks, School of Physics and Astronomy, Shanghai Jiao Tong University, Shanghai, 200240 China

**Keywords:** Optical sensors, Transformation optics, Nonlinear optics

## Abstract

Given that spin and orbital angular momenta of photons have been widely investigated in optical communication and information processing systems, efficient decoding of optical vortex states using a single element is highly anticipated. In this work, a wavelength-independent holographic scheme has been proposed for total angular momentum sorting of both scalar and vector vortex states with a stationary broadband geometric-phase waveplate by means of reference-free shearing interferometry. The entangled spin and orbital angular momentum modes can be distinguished simultaneously based on the spin–orbit optical Hall effect in order to realize single-shot vortex detection. The viability of our scheme has also been demonstrated experimentally.

## Introduction

It has been established that photons can possess spin and orbital angular momenta (SAM and OAM)^1,2^. SAM is important in that it directly relates to the polarization of light in scalar and vector optical fields, which is an essential state variable in both classical and quantum optical systems^3,4^. OAM is associated with an optical vortex which gives rise to a twisted wavefront and such a structured field possesses a helical phase expressed as exp(*imϕ*), where *m* is the topological charge and *ϕ* is the azimuthal angle^5,6^. Because angular momentum degrees of freedom greatly expand the dimensions of light, both SAM and OAM have been widely investigated in the fields of optical communication, information processing and so on^7–11^. For example, a novel designed technique for multi-dimensional space/amplitude data coding has been proposed recently to realize optical communication^6^. Therefore, detecting such higher dimensional angular momentum states is fundamentally important, albeit challenging, in order to use this rich degree of freedom^12,13^.

Two most common and practical ways for vortex states decoding are pattern-matching and filtering methods. The pattern-matching method is widely utilized to directly image vortex states based on interference with a known reference wavefront or diffraction through specific apertures^14^. Through the filtering method, vortex beams are converted to Gaussian beams for angular momentum modes detection^15^. However, finding a coherent reference wave to decode unknown optical signals is difficult for practical applications. Besides, the interference or diffraction patterns of pattern matching may repeat periodically once OAM modes become larger, as reported by Zhao et al.^16^. The filtering method also has limitations because at least *N* filters are required for *N* vortex states, which sharply decreases the configurability of optical systems. Moreover, this method cannot be used to detect vector vortex states. With the appearance of on-chip plasmonic nanostructures^17^ and metasurfaces^18^, compact scalar and vector vortex detection strategies have been proposed. Although excellent progress in vortex decoding has been made, there is still extensive room for improving their optical performance. Specifically, restricted by the wavevector-matching condition, on-chip plasmonic nanogratings operate only at a specific wavelength. On the other hand, the detectable OAM modes of dielectric metasurfaces are generally no more than ten. Quite recently, to solve these problems, an azimuthal-quadratic metasurface has been presented for total angular momentum sorting^11^.

In this work, a wavelength-independent holographic scheme has been proposed to efficiently decode optical scalar and vector vortex states by means of geometric-phase-based shearing interferometry^19^. The entangled SAM and OAM modes are distinguished simultaneously based on the spin–orbit optical Hall effect to realize single-shot vortex detection. The properties of our method are listed as follows: (1) both scalar and vector vortex states in a large mode space can be decoded using a single broadband geometric-phase waveplate with the thickness of 1 μm and diffraction efficiency over 90% in order to improve the configurability of optical systems; (2) in view of the limits of conventional vortex detection techniques, simultaneous SAM and OAM distinguishment is mainly realized with metasurfaces, and thus we theoretically design and experimentally demonstrate another holographic method to achieve total angular momentum sorting efficiently through geometric-phase-based shearing interferometry, which could enrich the field of information processing; (3) the recording-erasing-recording cycles of the anisotropic pattern can be repeated over 100 times in the same sample and the structure of the fabricated geometric-phase waveplate is simplified while detection efficiency is maintained. We present the principle of vortex state decoding, theoretical simulations and experimental measurements to demonstrate the viability of our scheme.

## Results

### Principle of geometric-phase-based shearing interferometry

Since direct detection of scalar and vector vortex states is impossible using conventional detectors^11^, the holographic technique becomes a powerful tool to realize vortex states decoding. The conventional method of quantitative phase measurement through holographic interferometry is expressed as

1 $$ I(r,\phi ) = I_{1} (r,\phi ) + I_{2} (r,\phi ) + 2\sqrt {I_{1} (r,\phi )I_{2} (r,\phi )} \cos \left[ {\omega \tau + \Delta \psi (r,\phi )} \right] $$

in which (*r*, *ϕ*) are polar coordinates, *I*_*1*_ and *I*_*2*_ are the intensities of coaxial objective and reference beams, respectively, *ω* is optical angular frequency, and Δ*ψ* and *τ* are phase difference and time delay between two waves. To determine the phase item Δ*ψ*(*r*, *ϕ*), several measurements of *I*(*r*, *ϕ*) are generally required under different values of *ωτ*^20^. However, multiple measurements are not efficient and single-shot SAM and OAM sorting cannot be achieved. To overcome these problems, we fabricate a broadband geometric-phase waveplate to realize single-shot decoding of vortex states under different wavelength conditions.

Geometric phases in optics originate from the coupling between intrinsic angular momentum and rotation of coordinates^21^. When light carries angular momentum ***J*** and the coordinate system experiences rotations with an angular velocity of ***ω***_***ξ***_ defined with the coordinate parameter *ξ*, this *ξ*-evolution induces a geometric phase

2 $$ \Phi_{g} \left( \xi \right) = - \int {{\varvec{J}} \cdot {\varvec{\omega}}_{\xi } d\xi } . $$

By means of geometric-phase-based shearing interferometry, a primary (+) and a conjugate (−) waves carrying opposite geometric phases *Φ*_*g*±_(*r*, *ϕ*) are generated through the extrinsic Hall effect. The light intensity distribution within the holographic light field is^19^

3 $$ I(r,\phi ) = I_{ + } (r,\phi ) + I_{ - } (r,\phi ) + 2\sqrt {I_{ + } (r,\phi )I_{ - } (r,\phi )} \cos \left[ {\Delta \psi (r,\phi ) + \Delta \Phi_{g} \left( {r,\phi } \right)} \right], $$

where Δ*Φ*_*g*_(*r*, *ϕ*) is the geometric-phase gradient between the two diffraction beams. Here, we set the time delay equal to 0 for wavelength-independent detection. Due to the spatial modulation frequency of the geometric-phase gradient along *x*- and y-axes, geometric-phase-controlled holograms are formed within the overlapping area and the cross-term in Eq. (3) is applied for total angular momentum sorting.

**Figure 1:**
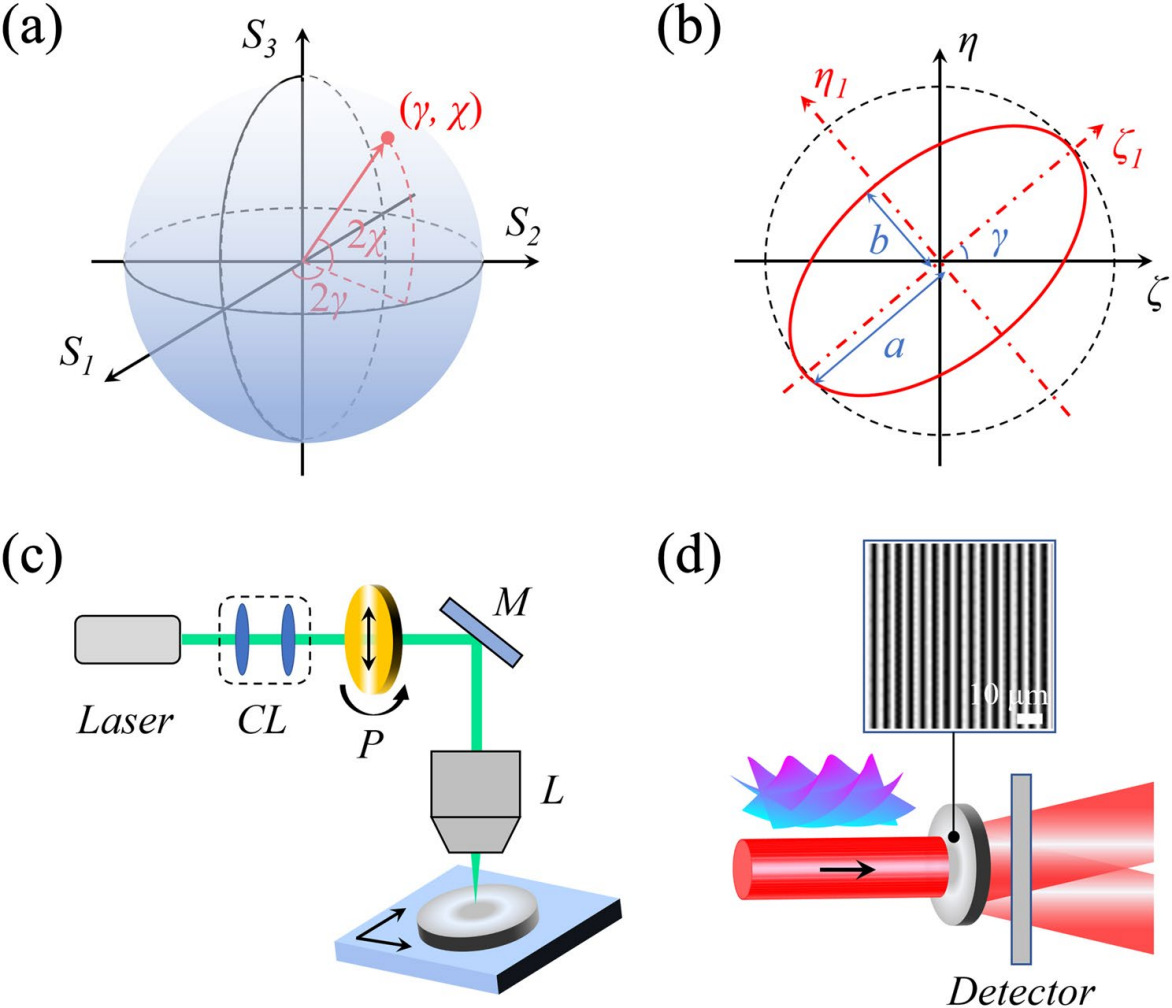
(**a**) Representation of optical polarization states on the Poincaré sphere. *γ* and *χ* represent the polarization direction and ellipticity, respectively. *S*_*1*_–*S*_*3*_ are the Stokes parameters. (**b**) Two-dimensional representation of polarization states through polarization ellipse. (**c**) Schematic of direct-writing fabrication of the designed broadband geometric-phase waveplate with the thickness of 1 μm. *CL* collimating lenses, *P* controlled linear polarizer, *M* mirror, *L* lens. (**d**) Experimental setup for efficient vortex detection using a single geometric-phase waveplate. The designed birefringence structure of the sample is detected experimentally with a polarized optical microscope.

Here, the design of the geometric-phase waveplate is discussed. In this work, a kind of photo-alignment liquid-crystalline films with the thickness of 1 μm is chosen as the sample because of the properties of strong photoinduced anisotropy and good thermal stability. The refractive-index matrix ***n*** of the film is

4 $$ {\varvec{n}} = {\varvec{n}}_{{\varvec{0}}} + {\varvec{n}}_{{\varvec{e}}} { = }n_{0} {\varvec{I}} + \left( {\begin{array}{*{20}c} {\overline{n} + \Delta n/2} & 0 \\ 0 & {\overline{n} - \Delta n/2} \\ \end{array} } \right), $$

where ***I*** is the identity matrix, $\overline{n} = \left( {\Delta n_{\parallel } + \Delta n_{ \bot } } \right)/2$ with $\Delta n_{\parallel }$ and $\Delta n_{ \bot }$ being the controlled refractive-index changes parallel and perpendicular to the polarization direction of the recording light, respectively, and Δ*n* is the photoinduced birefringence defined by the polarization dependence of refractive indices^22^. For a random optical polarization state on the Poincaré sphere in Fig. 1a, photoinduced refractive-index changes are $\Delta n_{\parallel } = \kappa_{a} E_{a}^{2} + \kappa_{b} E_{b}^{2}$ and $\Delta n_{ \bot } = \kappa_{b} E_{a}^{2} + \kappa_{a} E_{b}^{2}$ with *E*_*a*_ and *E*_*b*_ being the amplitudes of light components polarized along the directions of major- and minor-axes of the polarization ellipse in Fig. 1b, respectively, and *κ*_*a*_ and *κ*_*b*_ being the coefficients of photo-response. Therefore, the birefringence matrix in the *ζ*_*1*_-*η*_*1*_ coordinate system can be rewritten as

5 $$ {\varvec{n}}_{{\varvec{e}}} = \left( {\begin{array}{*{20}c} {\overline{\kappa } \left( {E_{a}^{2} + E_{b}^{2} } \right) + \Delta \kappa \left( {E_{a}^{2} - E_{b}^{2} } \right)} & 0 \\ 0 & {\overline{\kappa } \left( {E_{a}^{2} + E_{b}^{2} } \right) - \Delta \kappa \left( {E_{a}^{2} - E_{b}^{2} } \right)} \\ \end{array} } \right) $$

in which $\overline{\kappa } = \left( {\kappa_{a} + \kappa_{b} } \right)/2$ and $\Delta \kappa = \left( {\kappa_{a} - \kappa_{b} } \right)/2$. To obtain ***n***_***e***_ in the system of (*ζ*, *η*), the matrix in Eq. (5) is rotated through ${\varvec{n}}_{{\varvec{e}}} (\gamma ) = {\varvec{R}}( - \gamma ){\varvec{n}}_{{\varvec{e}}} {\varvec{R}}(\gamma )$ with ***R***(*γ*) being the rotation matrix^23^, and we obtain

6 $$ {\varvec{n}}_{{\varvec{e}}} \left( \gamma \right) = \left( {\begin{array}{*{20}c} {\overline{\kappa } S_{0} + \Delta \kappa S_{1} } & {\Delta \kappa S_{2} } \\ {\Delta \kappa S_{2} } & {\overline{\kappa } S_{0} - \Delta \kappa S_{1} } \\ \end{array} } \right), $$

where *S*_*0*_–*S*_*2*_ are the Stokes parameters of the recording light field^24^. Consequently, the Jones matrix of the recorded geometric-phase waveplate is calculated through ${\varvec{T}} = \exp \left[ {i2\pi \left( {{\varvec{n}}_{{\varvec{0}}} + {\varvec{n}}_{{\varvec{e}}} (\gamma )} \right)d/\lambda_{r} } \right]$ with *d* being the thickness of the sample and *λ*_*r*_ being the wavelength of the recording light^25^.

In order to realize total angular momentum sorting, the Stokes vector of the recording light field is controlled as

7 $$ \left( {\begin{array}{*{20}c} {S_{0} } \\ {S_{1} } \\ {S_{2} } \\ \end{array} } \right) = \frac{{\lambda_{r} }}{4d\Delta \kappa }\left( {\begin{array}{*{20}c} 1 \\ {\cos \left[ {2\gamma (r\cos \phi )} \right]} \\ {\sin \left[ {2\gamma (r\cos \phi )} \right]} \\ \end{array} } \right), $$

where *γ*(*r*cos*ϕ*) is space-dependent to encode desired geometric phase profiles in diffraction beams. By means of the all-optical direct-writing system with three degrees of freedom in Fig. 1c (two for spatial scanning and one for polarizer control), the photoinduced birefringence pattern described by Eqs. (6) and (7) is recorded and stored in the sample. The orientations of liquid crystals are controlled by the polarization direction of the recording light and the polarization direction is manipulated to change periodically in space with the polarizer. The structure of the all-optically fabricated geometric-phase waveplate is detected experimentally with a polarized optical microscope, as demonstrated in Fig. 1d. The Jones matrix of the geometric-phase waveplate is calculated as

8 $$ {\varvec{T}}_{{\mathbf{ \pm }}} = \frac{i}{2}\left( {\begin{array}{*{20}c} {\exp \left[ { \pm 2i\gamma \left( {r\cos \phi } \right)} \right]} & { \pm i\exp \left[ { \pm 2i\gamma \left( {r\cos \phi } \right)} \right]} \\ { \pm i\exp \left[ { \pm 2i\gamma \left( {r\cos \phi } \right)} \right]} & { - \exp \left[ { \pm 2i\gamma \left( {r\cos \phi } \right)} \right]} \\ \end{array} } \right), $$

which performs the shearing by writing opposite geometric phases on the two angular momentum components of the incident wavefront. For normal incidence, the diffraction efficiency is measured 92.4%. The spin-dependent geometric-phase gradient between the two diffraction beams is $\Delta \Phi_{g} = 2\Delta \sigma \gamma (r\cos \phi )$ with $\Delta \sigma = \sigma_{ + } - \sigma_{ - }$ being the SAM difference. This geometric-phase gradient produces a transverse component in the momentum of light that is generated by the coordinate gradient of the geometric phase with spatial modulation frequency and the refractive-index gradient in the sample plays the role of the external driving force to induce the birefringence-induced Hall effect^21^, resulting in simultaneous angular momentum distinguishment. The primary and conjugate diffraction beams interfere and a detector is applied to determine the spatial coordinates within the holographic light field, as shown in Fig. 1d. The vortex state is one-to-one corresponding to the same spatial coordinate under different wavelength conditions.

### Single-shot detection of scalar vortex states

Considering a single-ringed Laguerre–Gaussian beam, the Jones vector of the incident light is

9 $$ {\varvec{U}}_{{\varvec{i}}} = U_{0} \left( {\begin{array}{*{20}c} {\cos \gamma \cos \chi - i\sin \gamma \sin \chi } \\ {\sin \gamma \cos \chi + i\cos \gamma \sin \chi } \\ \end{array} } \right)\exp \left( {im\phi } \right), $$

where $U_{0} \propto \left( {r/\omega_{0} } \right)^{m}$ with *ω*_*0*_ being the waist width and *m* being the OAM order^26^. The primary and conjugate diffraction beams in the holographic light field are expressed as

10 $$ \begin{aligned} U_{ + }^{\dag } & = U_{0} \frac{\sin \chi - \cos \chi }{2}\exp \left[ {2i\gamma (r\cos \phi )} \right]\exp \left[ {i(\gamma + m\pi - m\phi )} \right] \\ U_{ - }^{\dag } & = U_{0} \frac{\sin \chi + \cos \chi }{2}\exp \left[ { - 2i\gamma (r\cos \phi )} \right]\exp \left[ {i(\gamma + m\phi )} \right]. \\ \end{aligned} $$

**Figure 2:**
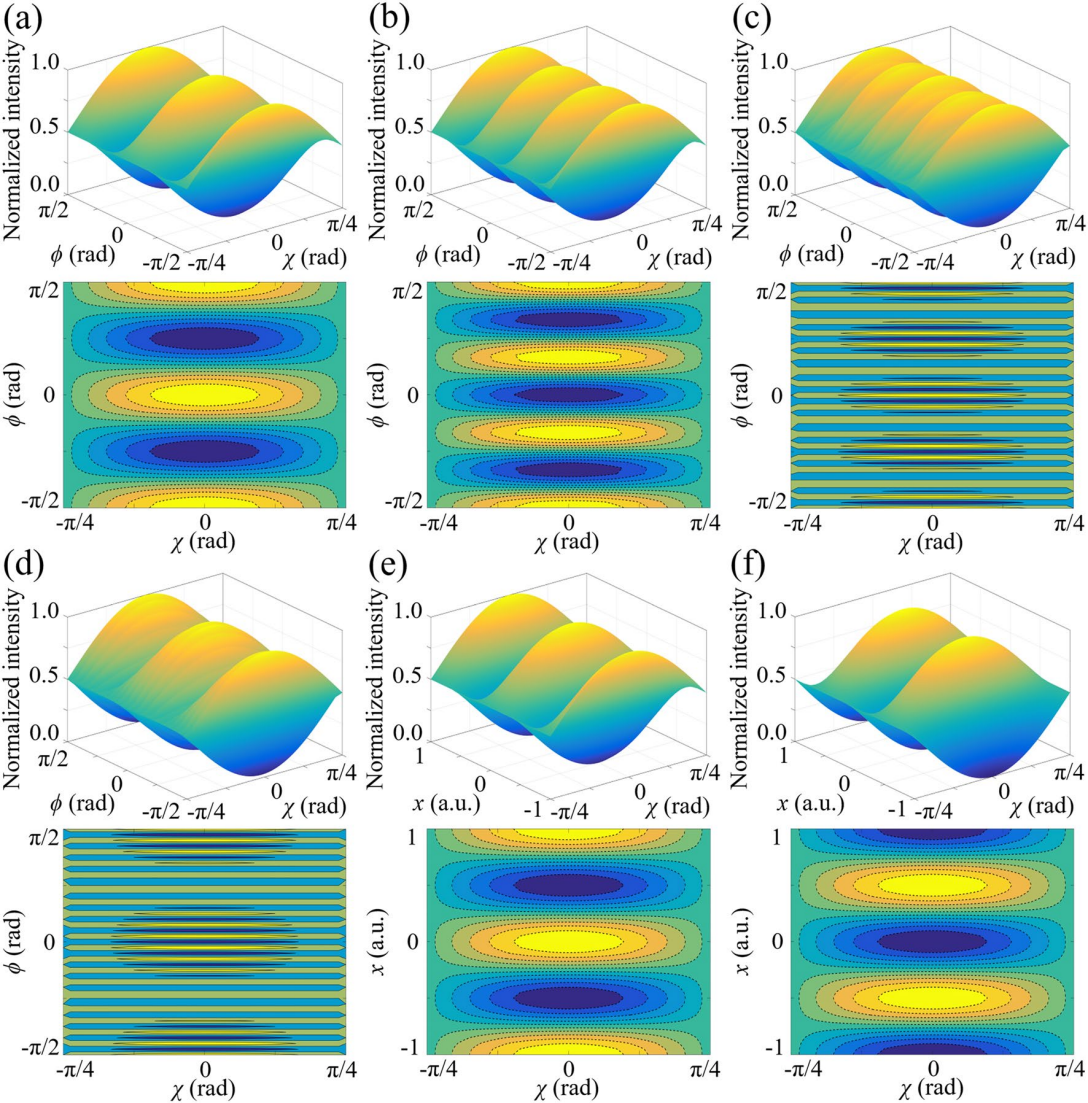
Changing behaviors of interference light intensities along the azimuthal direction when *χ* of photons varies between − π/4 and π/4 in terms of the OAM states of (**a**) *m* = 2, (**b**) *m* = 3, (**c**) *m* = 18 and (**d**) *m* = 19. The light intensities along *x*-axis (*r*cos*ϕ*) are controlled to change sinusoidally by the SAM magnitude when the topological charges are (**e**) even and (**f**) odd, regardless of the value of OAM. Contour maps are presented below each three-dimensional diagram.

The influence of the SAM component of scalar vortex states on the intensity distribution of interference light are simulated in Fig. 2. According to Fig. 2a, when the topological charge of the incident light equals 2, the light intensity varies periodically with the azimuthal angle because of the formation of the geometric-phase-induced interference fringes. The peaks of light intensity are obtained at *ϕ* = 0 and *ϕ* =  ± π/2 whatever the SAM is. On the other hand, the light intensity changes sinusoidally from 0 to the local extremum value and back to 0 as *χ* is controlled between − π/4 and π/4, regardless of the azimuthal angle. When *m* = 3 in Fig. 2b, the light intensity keeps maximum when *ϕ* is equal to ± π/2, while that decreases to the minimum value at the location of *ϕ* = 0. In terms of higher OAM states in Fig. 2c, d where *m* = 18 and 19, large topological charges result in densification of interference fringes, while the visibility of the inference fringes is solely controlled by *χ* and thus our scheme keeps efficient. On the other hand, when *ϕ* = 0, the light intensity also varies periodically along *x*-axis (*r*cos*ϕ*) and the intensity pattern is reversed when the topological charge changes from odd to even numbers, while the period is independent on SAM or OAM of photons, as presented in Fig. 2e, f, because the fringe spacing along *x*-axis is only determined by the spatial modulation frequency of the geometric-phase gradient.

**Figure 3:**
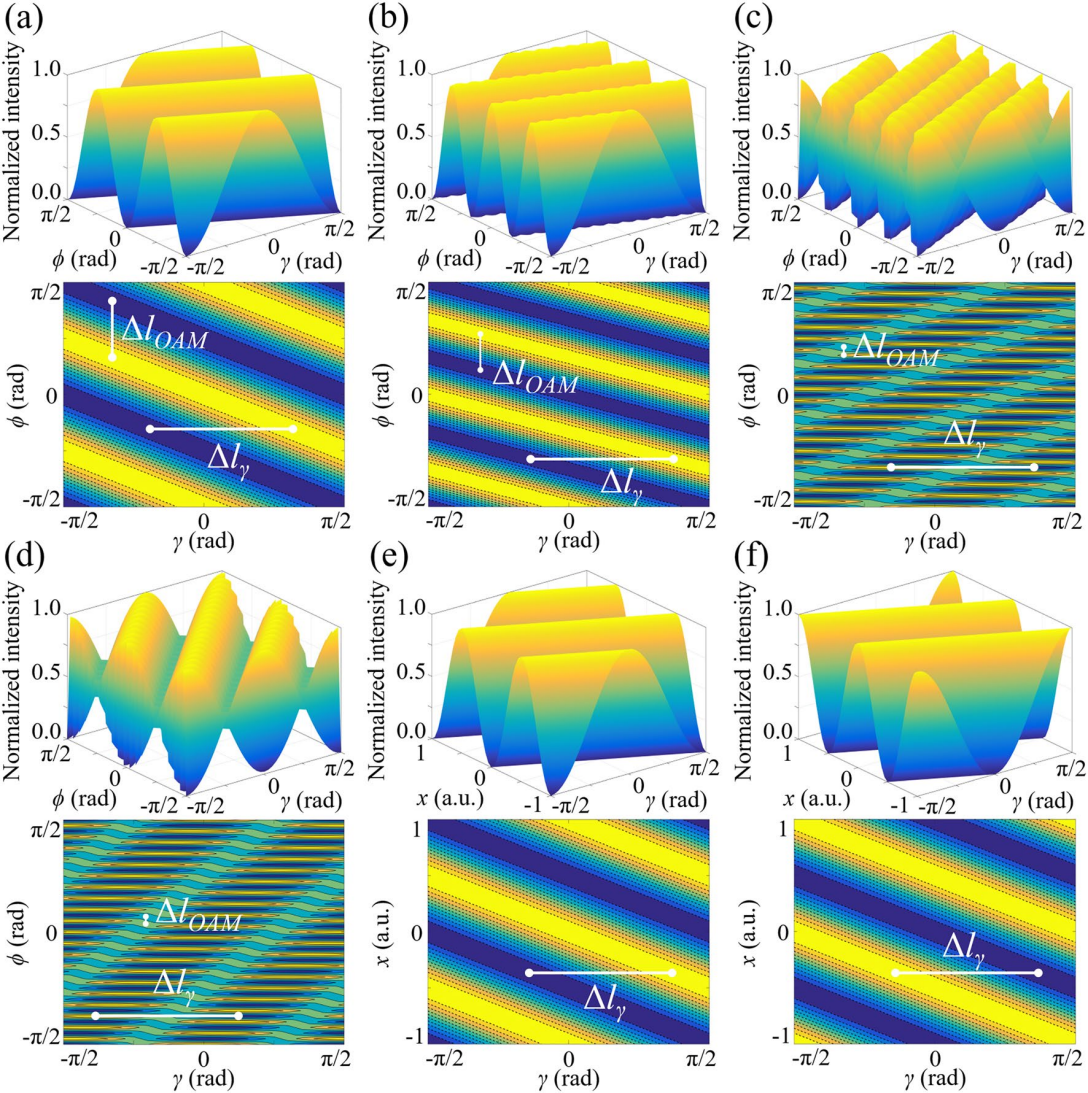
Dependence of light intensities on the azimuthal angle and polarization direction when the topological charges are (**a**) *m* = 2, (**b**) *m* = 3, (**c**) *m* = 18 and (**d**) *m* = 19. As the topological charges change from (**e**) even numbers to (**f**) odd numbers, the light intensity along *x*-axis is reversed because of a further π phase shift. The lengths of Δ*l*_*γ*_ keep constant in (**a**–**f**).

The dependence of light intensity distribution on the polarization direction of incident light is calculated and the simulated results are presented in Fig. 3. According to Fig. 3a–d, the light intensity not only changes periodically with the azimuthal angle but also is modulated from maximum to minimum and back to maximum by *γ* along the azimuthal direction when *m* = 2, 3, 18 and 19. On the other hand, according to Fig. 3e, f, the light intensity distribution is also reversed under the conditions of odd and even topological charges along *x*-axis. Different from Fig. 2e, f where *χ* controls the visibility of the holographic light field, a *γ*-dependent displacement of the geometric-phase-based interference fringes is induced. As demonstrated in Fig. 3e, f, when the polarization direction is rotated at a fixed angle Δ*γ*, the interference pattern displacement Δ*l*_*γ*_ along *x*-axis keeps constant for different OAM states. Figure 3a–d shows that Δ*l*_*γ*_ are still equal to those in Fig. 3e, f (half of the period of interference fringes on *x*-axis) in terms of different azimuthal angles, and thus this transverse spatial displacement is only along *x*-axis and the distance is determined by the polarization direction.

**Figure 4:**
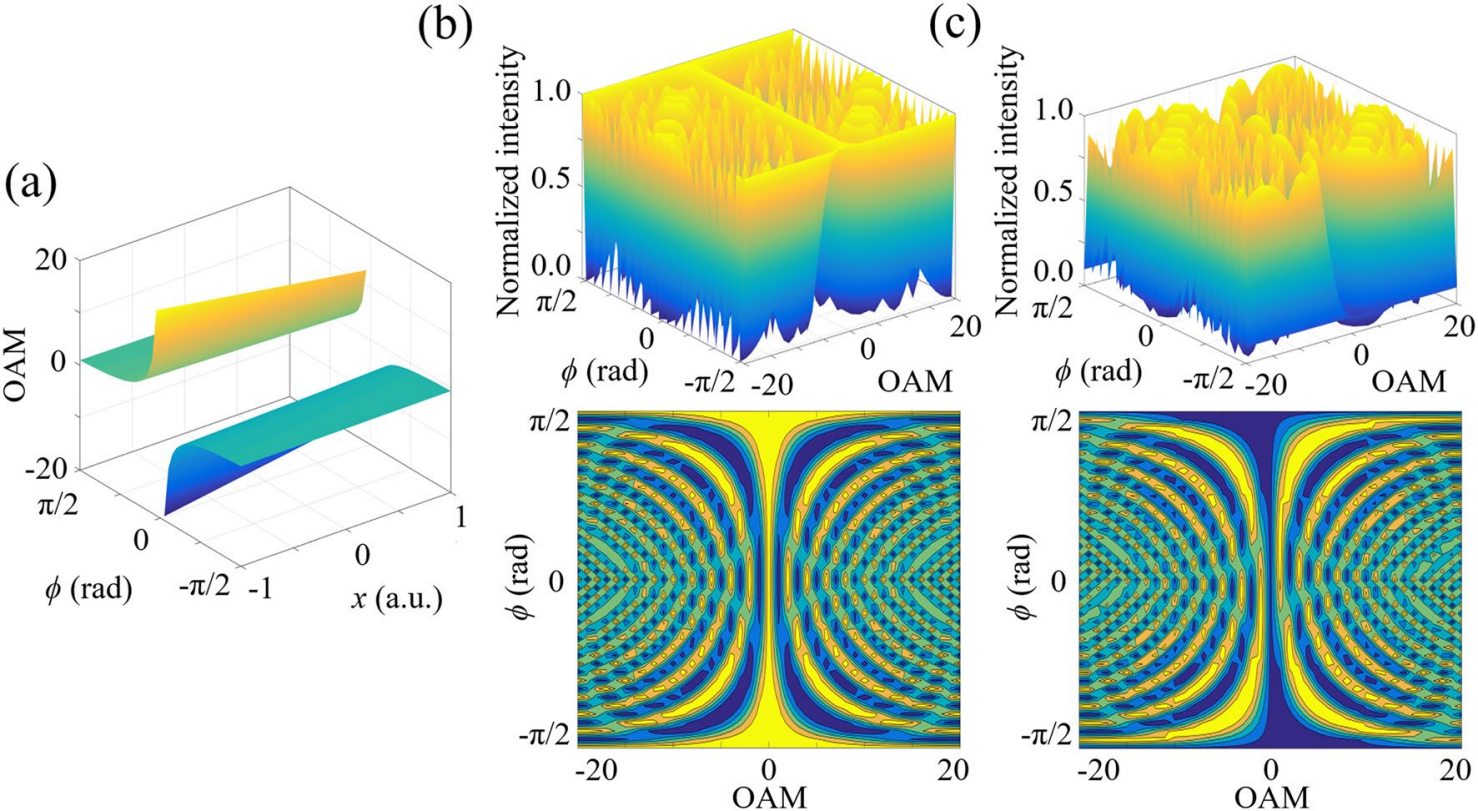
(**a**) OAM detection by means of geometric-phase-based shearing interferometry. (**b**) Simulated result of light intensity variation along the azimuthal direction in terms of various OAM states. With the change of the topological charge, an OAM-dependent displacement of interference fringes is induced along the azimuthal direction. (**c**) Changing behaviors of light intensities along *x*-axis become asymmetric for *m* > 0 and *m* < 0, which is applied to determine the sign of OAM states.

The magnitude and sign of OAM states can both be detected simultaneously. For a fixed azimuthal angle, the value of topological charges is one-to-one corresponding to the spatial coordinate of the *n*th-order interference stripe, as demonstrated in Fig. 4a. Moreover, with increasing the range along *x*-axis, the measurable value of topological charges becomes larger and is not limited between -20 and 20. According to Fig. 4b, alternate variation of light intensity along the azimuthal direction is induced with the increase of the topological charge. This result is attributed to the OAM-dependent displacement of the geometric-phase-based interference fringes. Different from the SAM-induced displacement along *x*-axis in Fig. 3, the OAM-induced displacement is in the azimuthal direction. Figure 3a–d present that for a fixed polarization direction, the stripe spacing Δ*l*_*OAM*_ in *y*-direction becomes smaller as the OAM state is modulated from *m* = 2 to *m* = 19, and thus the component of OAM-dependent displacement along *y*-axis is related to the absolute value of topological charge. Furthermore, along *x*-axis in Fig. 4c, the light intensity variation becomes asymmetric for *m* > 0 and *m* < 0, which is applied to determine the sign of OAM.

**Figure 5:**
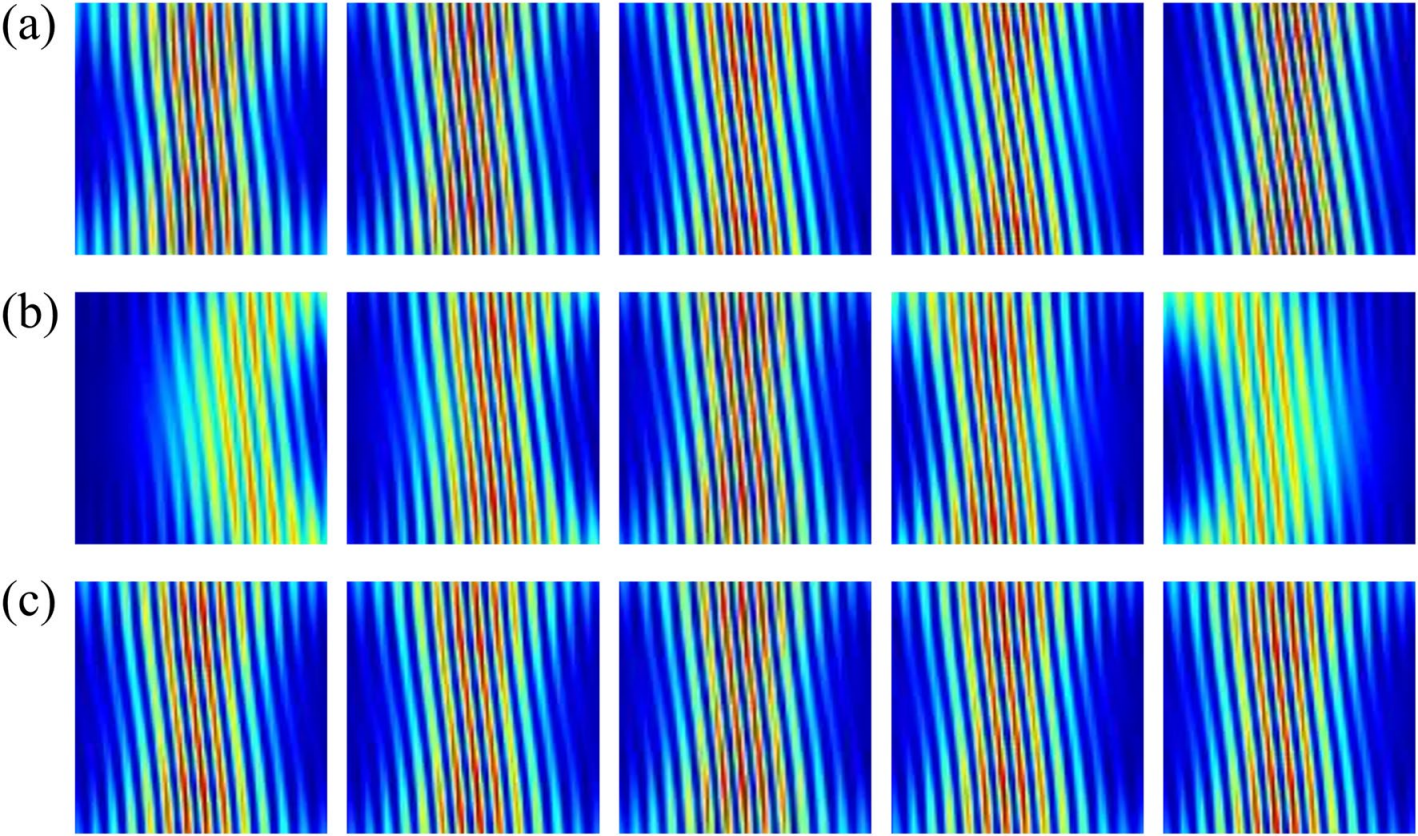
Experimentally detected holograms induced by geometric-phase-based shearing interferometry are presented. From left to right: (**a**) the OAM order increases from 2 to 6; (**b**) *γ* = − π/3, − π/6, 0, π/6 and π/3 with *m* = 3; (**c**) *χ* = − π/6, − π/12, 0, π/12 and π/6 with *m* = 3.

**Figure 6:**
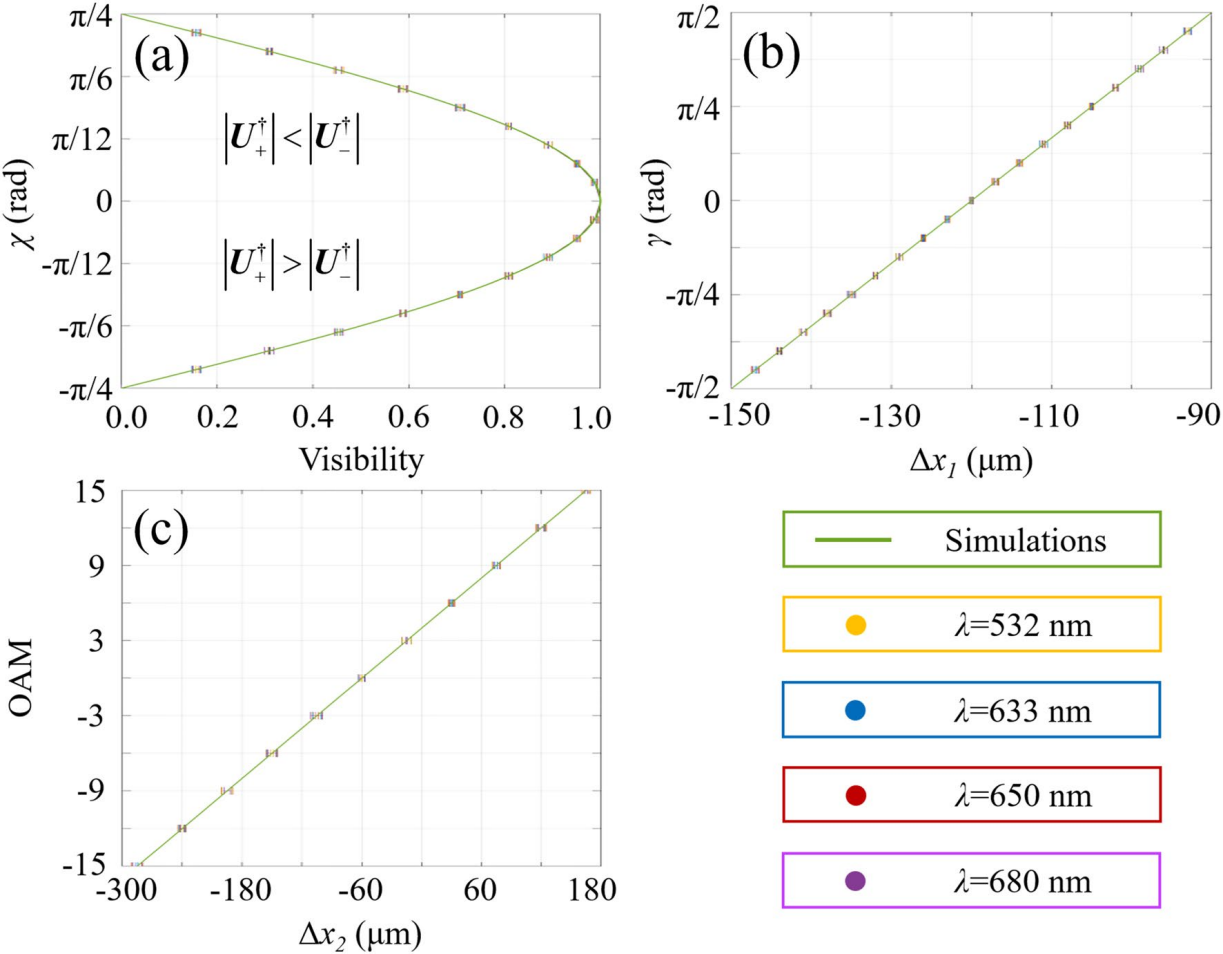
Theoretical simulations (curves) and experimental results (error bars) of single-shot decoding of scalar vortex states under various wavelength conditions.

Here, the viability of our scheme is demonstrated experimentally. In this work, the spatial modulation frequency of the geometric-phase gradient is set as 3 × 10^5^ m^−1^ and a 12 × zoom lens is applied for the detector. The SAM of scalar vortex states can be obtained through the normalized Stokes parameters $s_{1} = \cos \left( {2\gamma } \right)\cos \left( {2\chi } \right)$, $s_{2} = \sin \left( {2\gamma } \right)\cos \left( {2\chi } \right)$ and $s_{3} = \sin \left( {2\chi } \right)$ on the Poincaré sphere. The OAM components of scalar vortex states are decoded through the value of *m*. Comparing with the simulated results and experimental data in Figs. 5 and 6, single-shot wavelength-independent quantitative measurement of scalar vortex states has been realized through determining the coordinates of the location of the zeroth-order interference stripe on *x*-axis (*r* = *x*_*1*_, *ϕ* = 0) and the location of the + first-order interference stripe at the azimuthal angle of π/4 (*r*=$\sqrt{2}$ *x*_*2*_, *ϕ* = π/4).

### Angular momentum sorting of vector vortex states

**Figure 7:**
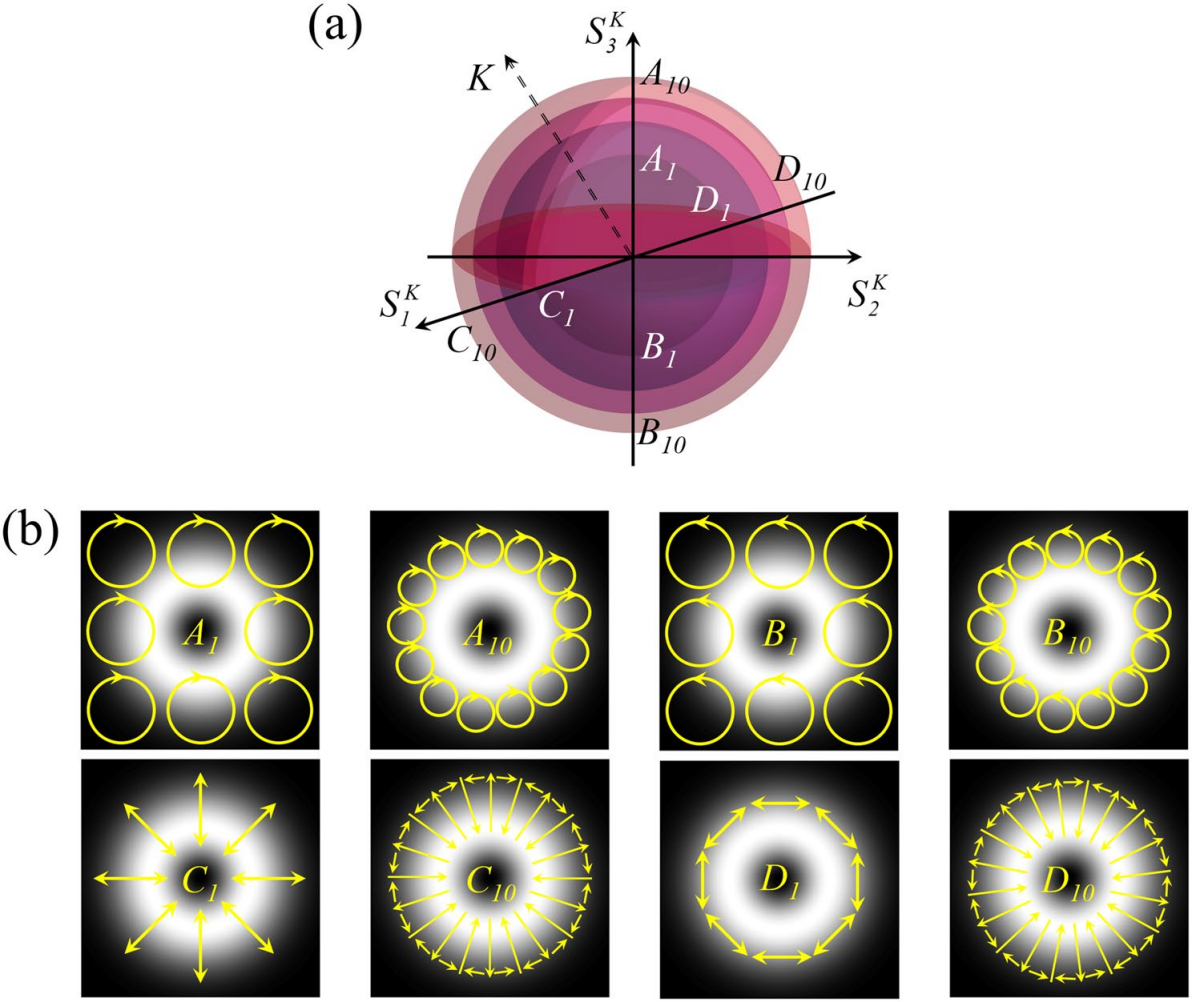
(**a**) Representation of vector optical fields on multi-layered higher-order Poincaré spheres. Each layer is corresponding to a certain value of *K*. (**b**) Simulation of local polarization vector states at various positions on the higher-order Poincaré sphere.

Different from scalar vortex states, vector optical fields cannot be described through the regular Poincaré sphere in Fig. 1a because of the spatially non-homogeneous polarization distribution. Therefore, higher-order Poincaré spheres should be introduced to describe the vector vortex states^7^, as shown in Fig. 7a. The mixed SAM states of phase vortices in the previous section is expressed in the space spanned by the eigenstates of SAM, left-circular $\left| L \right\rangle$ and right-circular polarization $\left| R \right\rangle$, i.e., $\hat{H}_{\sigma } = span\left\{ {\left| L \right\rangle ,\left| R \right\rangle } \right\}$^27^. Then we expand the space with the spatial modes of light $\hat{H}_{K}$ and the direct sum of vector spaces is

11 $$ \hat{H}_{V} = \oplus \hat{H}_{\sigma ,K} . $$

To completely represent higher-order entangled angular momentum states, two OAM-carrying orthogonal bases with continuously variable ellipticity are defined as

12 $$ \begin{aligned} \left| {\xi_{N}^{K} } \right\rangle & = \frac{1}{\sqrt 2 }\left[ {\exp \left( { - i\delta } \right){\mathbf{e}}_{{\varvec{x}}} - i\exp \left( {i\delta } \right){\mathbf{e}}_{{\varvec{y}}} } \right]\exp \left( { - iK\phi } \right) \\ \left| {\xi_{S}^{K} } \right\rangle & = \frac{1}{\sqrt 2 }\left[ {\exp \left( { - i\delta } \right){\mathbf{e}}_{{\varvec{x}}} + i\exp \left( {i\delta } \right){\mathbf{e}}_{{\varvec{y}}} } \right]\exp \left( { + iK\phi } \right), \\ \end{aligned} $$

where 2*δ* is the phase difference between *x*- and *y*-components, and *K* is related to the OAM component of vector vortex states^28^. We choose *δ* = π/2 in this work and an arbitrary vector vortex state can be described by

13 $$ \left| {\psi^{K} } \right\rangle = \Omega_{N}^{K} \left| {\xi_{N}^{K} } \right\rangle + \Omega_{S}^{K} \left| {\xi_{S}^{K} } \right\rangle , $$

where $\Omega_{N}^{K} = \sin \alpha_{K} \exp \left( { - i\beta_{K} } \right)$ and $\Omega_{S}^{K} = \cos \alpha_{K} \exp \left( { + i\beta_{K} } \right)$ with $\alpha_{K} \in \left[ {0,\pi /2} \right]$ and $\beta_{K} \in \left[ { - \pi /2,\pi /2} \right]$. Referencing the definition of the Stokes parameters of regular Poincaré sphere, the higher-order Stokes parameters are extended to

14 $$ \left( {\begin{array}{*{20}c} {S_{0}^{K} } \\ {S_{1}^{K} } \\ {S_{2}^{K} } \\ {S_{3}^{K} } \\ \end{array} } \right) = \left( {\begin{array}{*{20}c} {\left| {\left\langle {{\xi_{N}^{K} }} \mathrel{\left | {\vphantom {{\xi_{N}^{K} } {\psi^{K} }}} \right. \kern-\nulldelimiterspace} {{\psi^{K} }} \right\rangle } \right|^{2} + \left| {\left\langle {{\xi_{S}^{K} }} \mathrel{\left | {\vphantom {{\xi_{S}^{K} } {\psi^{K} }}} \right. \kern-\nulldelimiterspace} {{\psi^{K} }} \right\rangle } \right|^{2} } \\ {2{\text{Re}} \left( {\left\langle {{\xi_{N}^{K} }} \mathrel{\left | {\vphantom {{\xi_{N}^{K} } {\psi^{K} }}} \right. \kern-\nulldelimiterspace} {{\psi^{K} }} \right\rangle \left\langle {{\xi_{S}^{K} }} \mathrel{\left | {\vphantom {{\xi_{S}^{K} } {\psi^{K} }}} \right. \kern-\nulldelimiterspace} {{\psi^{K} }} \right\rangle } \right)} \\ {2{\text{Im}} \left( {\left\langle {{\xi_{N}^{K} }} \mathrel{\left | {\vphantom {{\xi_{N}^{K} } {\psi^{K} }}} \right. \kern-\nulldelimiterspace} {{\psi^{K} }} \right\rangle \left\langle {{\xi_{S}^{K} }} \mathrel{\left | {\vphantom {{\xi_{S}^{K} } {\psi^{K} }}} \right. \kern-\nulldelimiterspace} {{\psi^{K} }} \right\rangle } \right)} \\ {\left| {\left\langle {{\xi_{N}^{K} }} \mathrel{\left | {\vphantom {{\xi_{N}^{K} } {\psi^{K} }}} \right. \kern-\nulldelimiterspace} {{\psi^{K} }} \right\rangle } \right|^{2} - \left| {\left\langle {{\xi_{S}^{K} }} \mathrel{\left | {\vphantom {{\xi_{S}^{K} } {\psi^{K} }}} \right. \kern-\nulldelimiterspace} {{\psi^{K} }} \right\rangle } \right|^{2} } \\ \end{array} } \right), $$

with Re() and Im() representing the real and imaginary parts^29^. Here $\chi \to \chi_{K} = \alpha_{K} - \pi /4$ and $\gamma \to \gamma_{K} = \beta_{K}$ to satisfy the normalized higher-order Stokes parameters $s_{1}^{K} = \cos \left( {2\gamma_{K} } \right)\cos \left( {2\chi_{K} } \right)$, $s_{2}^{K} = \sin \left( {2\gamma_{K} } \right)\cos \left( {2\chi_{K} } \right)$ and $s_{3}^{K} = \sin \left( {2\chi_{K} } \right)$. Several vector vortex states on higher-order Poincaré spheres are presented in Fig. 7b.

Though vector vortex light also combines polarization and spatial modes, the detection of vector vortex states is rather more complex than scalar vortex states because the spatial and polarization degrees of freedom of this form of structured light are coupled in a non-separable manner^30,31^. Through the same holographic method, the generated primary and conjugate waves based on the birefringence-induced spin–orbit optical Hall effect^32^ are expressed as

15 $$ \begin{aligned} & \left| {\psi_{ + }^{K} } \right\rangle = i\cos \alpha_{K} \exp \left[ { + 2i\gamma (r\cos \phi )} \right]\exp \left( {i\beta_{K} + 2iK\phi } \right)\left| {\xi_{N}^{K} } \right\rangle \\ & \left| {\psi_{ - }^{K} } \right\rangle = i\sin \alpha_{K} \exp \left[ { - 2i\gamma (r\cos \phi )} \right]\exp \left( { - i\beta_{K} - 2iK\phi } \right)\left| {\xi_{S}^{K} } \right\rangle . \\ \end{aligned} $$

By means of determining the coordinates of several positions within the holographic light field, single-shot decoding of vector vortex states (*K*, *γ*_*K*_, *χ*_*K*_) can be realized.

**Figure 8:**
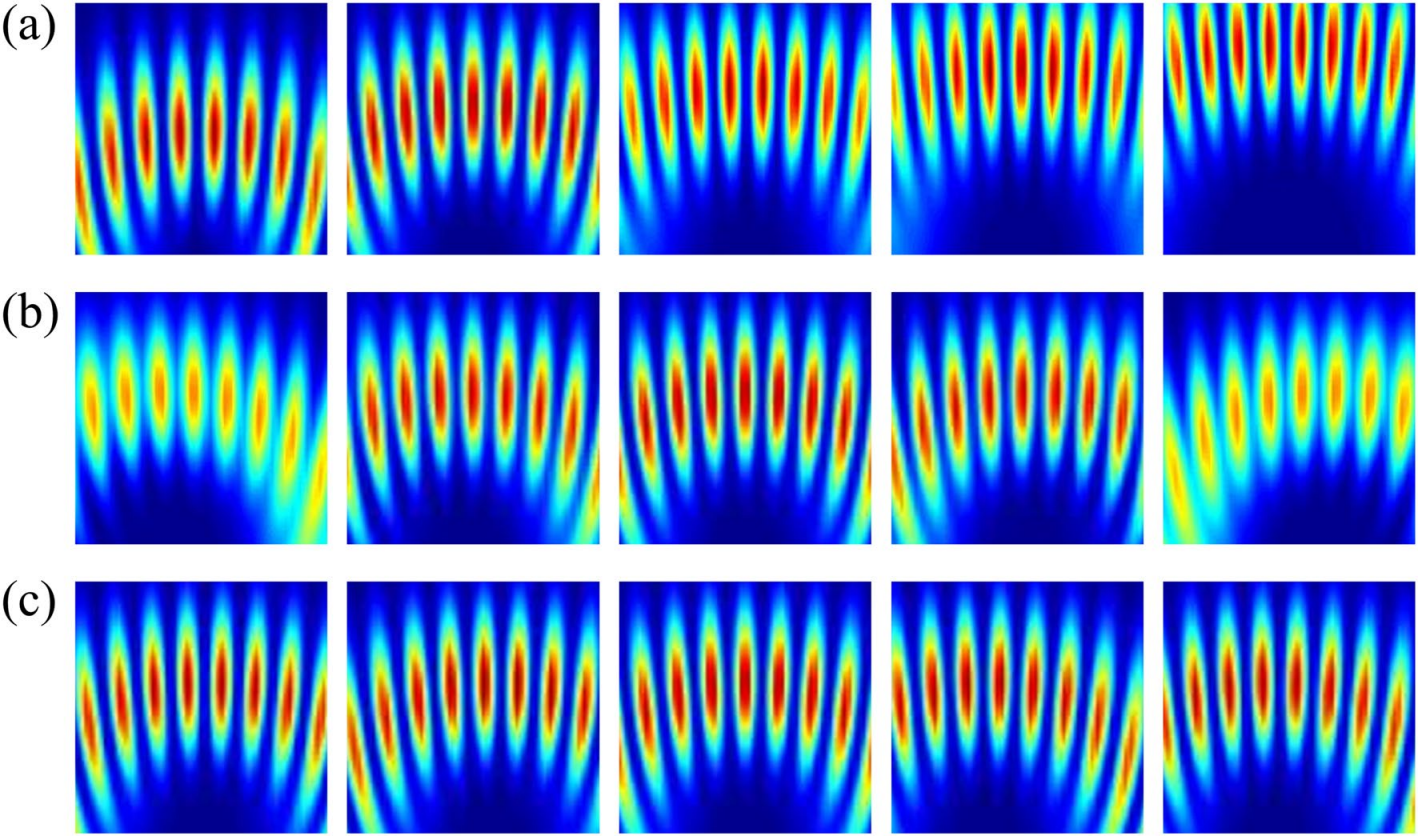
Experimentally detected holograms for vector vortex decoding are demonstrated. From left to right: (**a**) *K* increases from 2 to 6; (**b**) *γ*_*K*_ = − π/3, − π/6, 0, π/6 and π/3 with *K* = 3; (**c**) *χ*_*K*_ = − π/6, − π/12, 0, π/12 and π/6 with *K* = 3.

**Figure 9:**
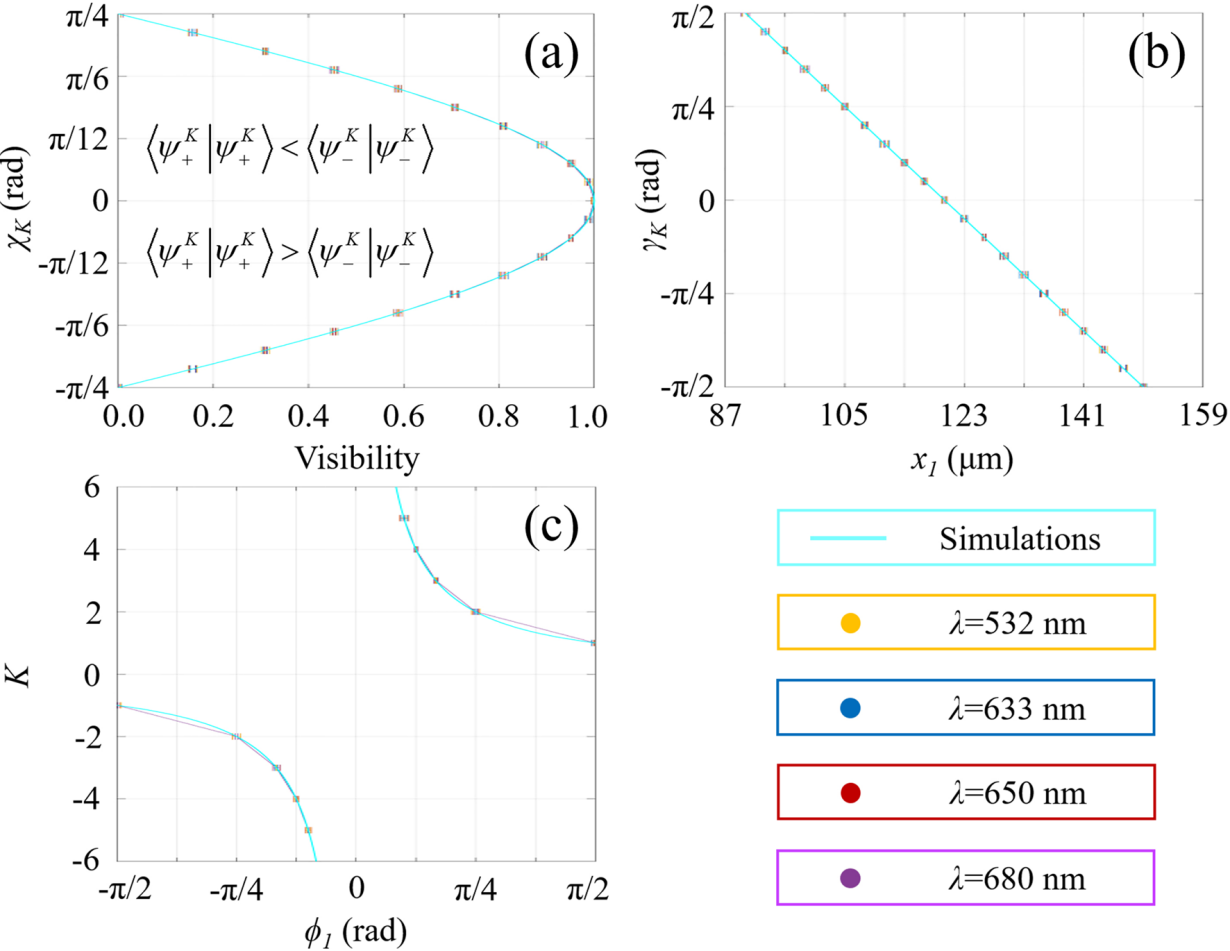
Theoretical simulations (curves) and experimental results (error bars) of single-shot detection of vector vortex states. Total angular momentum sorting under different conditions of wavelengths keeps the same in terms of the proposed scheme.

Theoretical simulations and experimental results on vector field detection are presented in Figs. 8 and 9. All vector vortex states are one-to-one corresponding to unique spatial coordinates without repeating. Two coordinates, (*r* = *x*_*1*_, *ϕ* = 0) and (*r* = *x*_*1*_/cos*ϕ*_*1*_, *ϕ* = *ϕ*_*1*_), are determined at the locations of the second-order bright interference stripe and the adjacent dark stripe, respectively. The normalized higher-order Stokes parameters are obtained through *χ*_*k*_ and *γ*_*k*_, and the vector vortex state can be represented on the *K*th-order Poincaré spheres. As we change the wavelength, the spatial coordinates corresponding to vector vortex states keep constant. There is a good agreement between the theoretical analysis and experimental measurements. Consequently, single-shot decoding of vector vortex states on higher-order Poincaré sphere has also been demonstrated experimentally by means of geometric-phase-based shearing interferometry.

## Discussion

In conclusion, single-shot wavelength-independent decoding of scalar and vector vortex states has been demonstrated both theoretically and experimentally by means of shearing interferometry using a single stationary geometric-phase waveplate. The birefringence-induced spin–orbit optical Hall effect is applied to divide the optical vortex states into several angular momentum components in order to realize simultaneous SAM and OAM sorting, which is now mainly realized through metasurfaces. Based on conventional detection techniques, the method on geometric-phase-based shearing interferometry is applicable within a certain wavelength range with the diffraction efficiency of 92.4% and the size of the optical element is beneficial to the configurability of optical systems. Comparing with recent works on vortex detection, the fabrication process and structure of the recorded geometric-phase waveplate are designed to become simpler, while it maintains detection functions and working efficiency. Moreover, SAM and OAM modes of different vortices in a large mode space have mainly been distinguished simultaneously through metasurfaces so far. In order to enrich the field of information decoding, another holographic method has been theoretically designed and experimentally demonstrated in this work to realize total angular momentum sorting efficiently. This scalability may make the proposed decoding method have applications in the fields of optical communication and information processing.

## Methods

### Material preparation

The sample is a kind of supermolecule materials through ionic self-assembly of poly ionic liquid and azobenzene dyes. The preparation process has been reported in Ref.^33^. The charged polymer poly (1-butyl-vinylpyridinium bromide) is selected as the main chain, and the methyl orange dye is selected as the building unit. For the preparation of ionic self-assembly complex, 2 mg/ml poly ionic liquid aqueous solution is added to methyl orange aqueous solution at the molar charge ratio of 1:1. The precipitated complex is filtrated and washed several times with doubly distilled water, then dried in vacuum at 60 °C for 12 h. The thickness of the sample is 1 μm.

## Data Availability

The datasets generated or analysed during the current study are available from the corresponding author on reasonable request.

## References

[1] Acebal P, Carretero L, Blaya S (2021). Extraordinary spin to orbital angular momentum conversion on guided zone plates. Sci. Rep..

[2] Rafayelyan M, Brasselet E (2018). Spin-to-orbital angular momentum mapping of polychromatic light. Phys. Rev. Lett..

[3] Zhao Z (2020). Dynamic spatiotemporal beams that combine two independent and controllable orbital-angular-momenta using multiple optical-frequency-comb lines. Nat. Commun..

[4] Yang S, Huang M, Zhao Y, Zhang HP (2021). Controlling cell motion and microscale flow with polarized light fields. Phys. Rev. Lett..

[5] Fang X, Ren H, Gu M (2019). Orbital angular momentum holography for high-security encryption. Nat. Photonics.

[6] Zhu L, Wang A, Deng M, Lu B, Guo X (2021). Experimental demonstration of multiple dimensional coding decoding for image transfer with controllable vortex arrays. Sci. Rep..

[7] Lyu Z (2019). All-optically controlled beam splitting through asymmetric polarization-based holography. Opt. Lett..

[8] Li R, Cao L (2021). Complex wavefront sensing based on coherent diffraction imaging using vortex modulation. Sci. Rep..

[9] Ostrovsky E, Cohen K, Tsesses S, Gjonaj B, Bartal G (2018). Nanoscale control over optical singularities. Optica.

[10] Pabón D, Ledesma S, Rebón L (2020). High-dimensional states of light with full control of OAM and transverse linear momentum. Opt. Lett..

[11] Guo Y (2021). Spin-decoupled metasurface for simultaneous detection of spin and orbital angular momenta via momentum transformation. Light-Sci. Appl..

[12] Mirhosseini M, Malik M, Shi Z, Boyd RW (2013). Efficient separation of the orbital angular momentum eigenstates of light. Nat. Commun..

[13] Nape I (2021). Measuring dimensionality and purity of high-dimensional entangled states. Nat. Commun..

[14] Berkhout GCG, Beijersbergen MW (2008). Method for probing the orbital angular momentum of optical vortices in electromagnetic waves from astronomical objects. Phys. Rev. Lett..

[15] Deng D, Zhao H, Han Y, Liu Y, Li Y (2021). Extending the detection range of optical vortices by dense phase stitching algorithm. J. Lightwave Technol..

[16] Zhao Q, Dong M, Bai Y, Yang Y (2020). Measuring high orbital angular momentum of vortex beams with an improved multipoint interferometer. Photonics Res..

[17] Feng F, Si G, Min C, Yuan X, Somekh M (2020). On-chip plasmonic spin-Hall nanograting for simultaneously detecting phase and polarization singularities. Light-Sci. Appl..

[18] Zhang S (2020). Broadband detection of multiple spin and orbital angular momenta via dielectric metasurface. Laser Photonics Rev..

[19] Alemán-Castaneda LA, Piccirillo B, Santamato E, Marrucci L, Alonso MA (2019). Shearing interferometry via geometric phase. Optica.

[20] Park Y, Depeursinge C, Popescu G (2018). Quantitative phase imaging in biomedicine. Nat. Photonics.

[21] Bliokh KY, Rodríguez-Fortuño FJ, Nori F, Zayats AV (2015). Spin-orbit interactions of light. Nat. Photonics.

[22] Shi Z (2020). Continuous angle-tunable birefringence with freeform metasurfaces for arbitrary polarization conversion. Sci. Adv..

[23] Dorrah AH, Rubin NA, Zaidi A, Tamagnone M, Capasso F (2021). Metasurface optics for on-demand polarization transformations along the optical path. Nat. Photonics.

[24] Marco D (2021). Customized depolarization spatial patterns with dynamic retardance functions. Sci. Rep..

[25] Nikolova, L. & Ramanujam, P. S. *Polarization Holography* (Cambridge University Press, 2011).

[26] Nobahar D, Khorram S, Rodrigues JD (2021). Vortex beam manipulation through a tunable plasma-ferrite metamaterial. Sci. Rep..

[27] Ndagano B (2017). A deterministic detector for vector vortex states. Sci. Rep..

[28] Liu Z (2017). Generation of arbitrary vector vortex beams on hybrid-order Poincaré sphere. Photonics Res..

[29] Ren Z-C (2015). Generalized Poincaré sphere. Opt. Express.

[30] Khan SN, Joshi S, Kanseri B, Senthilkumaran P (2021). Detection of partially coherent polarization singular vector beams using Stokes polarimetry. Appl. Phys. Lett..

[31] Chen Y (2020). Vector vortex beam emitter embedded in a photonic chip. Phys. Rev. Lett..

[32] Fu S (2019). Spin-orbit optical hall effect. Phys. Rev. Lett..

[33] Xiao S, Lu X, Lu Q (2007). Photosensitive polymer from ionic self-assembly of azobenzene dye and poly(ionic liquid) and its alignment characteristic toward liquid crystal molecules. Macromolecules.

